# A Discrete Fourier Transform-Based Signal Processing Method for an Eddy Current Detection Sensor †

**DOI:** 10.3390/s25092686

**Published:** 2025-04-24

**Authors:** Songhua Huang, Maocheng Hong, Ge Lin, Bo Tang, Shaobin Shen

**Affiliations:** 1 CGN Inspection Technology Co., Ltd., 191 Yangpu Road, Suzhou 215012, China; 2 National Engineering Research Center for Nuclear Power Plant Safety & Reliability, Suzhou 215012, China; 3 School of Electronic Science & Engineering, Southeast University, No. 2 Sipailou, Nanjing 211189, China

**Keywords:** eddy current sensor, discrete Fourier transform (DFT), non-destructive testing (NDT), signal processing, array eddy current sensor, spectrum leakage

## Abstract

This paper presents a discrete Fourier transform (DFT)-based signal processing framework for eddy current non-destructive testing (NDT), aiming to enhance signal quality for precise defect characterization in critical nuclear components. By enforcing strict periodicity matching between sampling points and signal frequencies, the proposed approach mitigates DFT spectrum leakage, validated via phase linearity analysis with errors of ≤0.07° across the 20 Hz–1 MHz frequency range. A high-resolution 24-bit analog-to-digital converter (ADC) hardware architecture eliminates complex analog balancing circuits, reducing system-wide noise by overcoming the limitations of traditional 16-bit ADCs. A 6 × 6 mm application-specific integrated circuit (ASIC) for array sensors enables three-dimensional (3D) defect visualization, complemented by Gaussian filtering to suppress vibration-induced noise. Our experimental results demonstrate that the digital method yields smoother signal waveforms and superior 3D defect imaging for nuclear power plant tubes, enhancing result interpretability. Field tests confirm stable performance, showcasing clear 3D defect distributions and improved inspection performance compared to conventional techniques. By integrating DFT signal processing, hardware optimization, and array sensing, this study introduces a robust framework for precise defect localization and characterization in nuclear components, addressing key challenges in eddy current NDT through systematic signal integrity enhancement and hardware innovation.

## 1. Introduction

**Figure 1:**
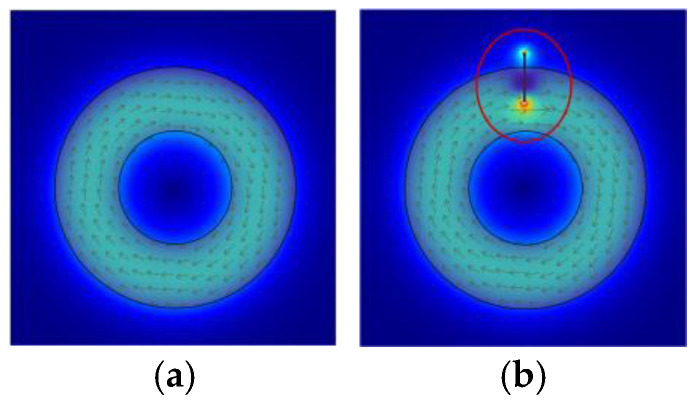
(**a**) Distribution of eddy currents when there are no defects in the conductor and (**b**) distribution of eddy currents when there is a crack in the conductor.

Eddy current testing, an NDT method rooted in electromagnetic induction, detects conductor defects by sensing magnetic field changes caused by disrupted eddy current trajectories [1,2]. Figure 1 illustrates the effect of defects on eddy current distribution; Figure 1a shows uniform trajectories in defect-free conductors, while Figure 1b depicts disrupted paths around a crack.

As illustrated in Figure 1, defects alter these trajectories, translating into measurable impedance variations in the detection sensor (induction coil). The sensor impedance Z is governed by material properties and test conditions, as in the following Equation (1) [3]:

*Z* = *F*(*ρ*,*μ*,*x*,*f*,*r*,*h*) (1)

where *ρ* (conductivity), *μ* (magnetic permeability), *f* (excitation frequency), *r* (probe radius), and *h* (sensor-specimen distance) define the testing framework, with *x* denoting material defects (e.g., wear, cracks, corrosion in nuclear heat transfer tubes). When other variables are fixed, *Z* directly reflects *x*, enabling defect characterization via impedance analysis.

**Figure 2:**
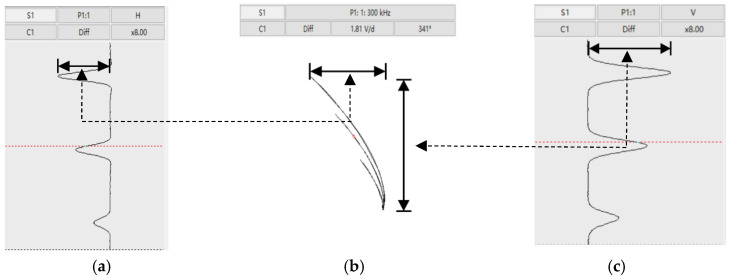
(**a**) Strip chart in the horizontal direction, showing changes in the real part of the sensor impedance; (**b**) impedance plan, characterizing the trajectory of sensor impedance changes; (**c**) strip chart in the vertical direction, showing changes in the imaginary part of the sensor impedance.

ECT signals are typically visualized as the real (resistance) and imaginary (reactance) components [2], with Figure 2 using strip charts to visualize impedance changes over time/position, derived from the impedance plane plot (Figure 2b) tracing impedance trajectories. The horizontal strip chart (Figure 2a) represents the real part signal (sensor resistance changes), while the vertical strip chart (Figure 2c) shows the imaginary part signal (sensor reactance changes).

The eddy current signal in Figure 2 is obtained at a frequency of 300 KHz. A critical aspect of ECT signal processing is frequency selection, balancing the skin effect with algorithm requirements. For nuclear applications using Bobbin probes, demanding 900 mm/s testing speed and 0.5 mm resolution, a sampling rate of 2000 Hz (with a safety margin) is set [4]. The discrete Fourier transform (DFT) algorithm, essential for extracting signal amplitude and phase, requires at least 10 signal cycles for noise suppression. When the sampling rate is set at 2000, it implies that there are at least 10 signal periods within 1/2000 s, thus dictating a minimum detection frequency of 20 kHz. This frequency constraint, fundamental for reliable defect analysis, becomes more complex with array sensors—a challenge that is systematically addressed in subsequent sections.

**Figure 3:**
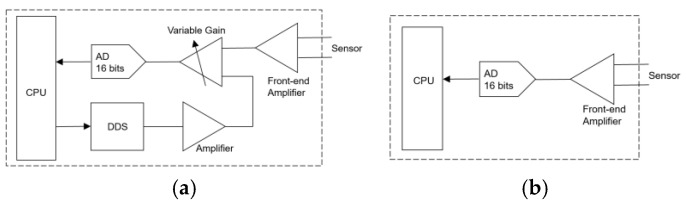
(**a**) Analog circuit signal processing method; (**b**) digital circuit signal processing method.

Recent decades have seen significant advancements in ECT signal processing, yet challenges persist in nuclear applications due to harsh environments (high temperature, radiation) and stringent requirements for defect resolution (0.5 mm) and testing speed (900 mm/s). Traditional analog-based systems, relying on 16-bit ADC and complex analog balancing circuits, suffer from limited dynamic range and high noise, often requiring real-time amplitude adjustment to avoid signal clipping. For example, the mainstream scheme (Figure 3a) uses direct digital frequency synthesis (DDS) with variable gain control to balance impedance mismatches, but this introduces electronic noise due to analog component tolerances, degrading spectral analysis accuracy [5].

In the digital domain, DFT has emerged as a key tool for extracting frequency-domain features, yet its application is hindered by spectrum leakage—spectral energy spreading across adjacent bins due to non-integer signal periods in the sampling window [6,7]. While prior studies have proposed windowing techniques to mitigate leakage, these methods often compromise phase linearity, which is critical for precise impedance analysis in nuclear ECT [8]. Additionally, array eddy current sensors, essential for 3D defect visualization in narrow-diameter tubes (e.g., 15.4 mm inner diameter), face challenges in miniaturization and signal integrity due to transient noise from channel switching and limited sampling efficiency.

Against this backdrop, this paper presents a robust framework integrating DFT-based signal processing, hardware optimization, and array sensing to address these gaps. By enforcing strict periodicity matching between sampling points and signal frequencies, the proposed method eliminates DFT spectrum leakage, achieving phase linearity errors of ≤0.07° across 20 Hz–1 MHz (validated via experimental analysis). A high-resolution 24-bit ADC architecture (Figure 3b) removes the need for analog balancing circuits, reducing system noise and enabling direct, full-range signal acquisition in harsh environments. A custom 6 × 6 mm application-specific integrated circuit (ASIC) for array sensors facilitates 3D defect visualization, complemented by Gaussian filtering to suppress vibration-induced noise. These innovations are systematically validated through experimental and field tests, demonstrating superior signal quality and defect characterization compared to conventional techniques.

## 2. Implementation of Eddy Current Sensor Signal Processing

This section introduces the eddy current sensor signal processing method in terms of the following five aspects: hardware architecture, algorithm implementation, spectrum leakage mitigation, algorithm verification, and comparative testing.

### 2.1. Hardware Architecture

Non-destructive testing of critical nuclear power plant components often employs multi-frequency eddy current detection to mitigate interference from strong electromagnetic signals generated by adjacent support plates [4]. This technique enables simultaneous inspection at two or more excitation frequencies, where the superposition of frequency-domain response signals in a mixing channel suppresses support plate noise and isolates defect-related impedance variations. For example, in steam generator heat transfer tube inspections, five frequencies are generally used simultaneously [4].

Figure 3 contrasts the hardware architectures of two eddy current signal processing approaches. The mainstream scheme (Figure 3a) relies on a 16-bit analog-to-digital converter (ADC), necessitating a probe balancing circuit to address inherent impedance mismatches in induction coils. This circuit, implemented via direct digital frequency synthesis (DDS) integrated with a variable gain control system, is critical due to the limited dynamic range of 16-bit ADCs: when detecting large initial signals, unprocessed inputs often exceed the ADC’s voltage range, requiring real-time amplitude adjustment to “rebalance” the signal to the origin.

In contrast, the proposed digital architecture (Figure 3b) utilizes a 24-bit ADC, completely eliminating the need for analog balancing circuitry. The 24-bit ADC provides a dynamic range 256 times wider than its 16-bit counterpart, enabling direct, full-range signal acquisition without hardware-level amplitude correction. This design circumvents the complexity of traditional balancing circuits—comprising DDS modules, operational amplifiers, and adjustable gain stages—which introduce significant electronic noise due to analog component tolerances and signal chain imperfections.

Removing analog balancing components offers the following dual benefits: (1) a significant reduction in electronic noise, enhancing signal integrity for spectral analysis; and (2) lower power consumption and heat dissipation due to fewer active analog components, improving system reliability in high-temperature, high-radiation nuclear environments. This hardware optimization forms the basis for a robust, low-noise signal acquisition platform compatible with the wide-frequency requirements of array-based eddy current sensors.

### 2.2. Algorithm Implementation

**Figure 4:**
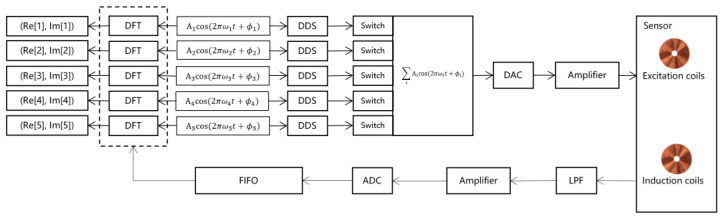
Digitization processing method of eddy current sensor signals.

The eddy-current signal digital processing framework, illustrated in Figure 4, is realized through an ARM + FPGA hardware architecture. The ARM processor manages host–computer interaction (e.g., receiving configuration parameters and uploading detection data), while the FPGA executes real-time control of excitation signal generation and detection signal extraction. The system includes excitation coils to induce electromagnetic fields and induction coils to capture impedance variations indicative of defects.

1. Digitization of excitation signals

Customized multi-frequency excitation signals are generated based on the material properties and defect characteristics of the inspected object. As demonstrated in Figure 4, up to five distinct frequencies (e.g., 20 kHz, 100 kHz, 300 kHz, 400 kHz, and 550 kHz for Bobbin probes [2]) can be configured simultaneously, with independent control over frequency ($\omega_{i}$), phase ($\varphi_{i}$), and amplitude ($A_{i}$). DDS technology converts these parameters into high-precision digital sine waves by sampling the waveform at fine phase intervals and constructing a phase-amplitude lookup table [5], ensuring exceptional stability, resolution, and wide-range tunability.

**Figure 5:**
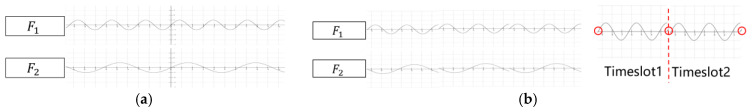
(**a**) Continuous sinusoidal signals; (**b**) repeated sinusoidal excitation signals.

Figure 5 provides a comparison of two excitation signal formats, namely continuous sinusoids (Figure 5a) versus periodic repetitive signals (Figure 5b). For traditional continuous sinusoids, based on the set eddy-current signal sampling rate ($f_{s}$), these signals are typically truncated into timeslots (T = 1/$f_{s}$) for sampling in a manner that often does not align with an integer number of signal periods, leading to phase discrepancies across slots and non-reproducible detection signals for identical defects. The proposed method uses DDS to generate stable repetitive signals (Figure 5b), ensuring that each time slot contains an exact integer number of signal cycles. This synchronization guarantees consistent ADC sampling values for the same defect, significantly enhancing signal processing repeatability.

2. Excitation signal and detection signal processing

Digitized sinusoidal signals are mathematically superimposed as $\sum_{i}A_{i}\cos(2\pi \omega_{i}t+\phi_{i})$. For wide-frequency applications, peak amplitude overlap during superposition can cause signal distortion. Thus, configuration parameters are constrained to ensure the combined amplitude of all frequency components does not exceed the maximum allowable range. The digital signal is then converted to an analog waveform via a digital-to-analog converter (DAC), and a power amplifier boosts the signal strength to effectively excite induction coils, compensating for the DAC’s limited drive capability.

3. DFT (Discrete Fourier Transform)

DFT serves as the core of the signal analysis framework, extracting frequency-domain features from time-domain signals. After amplification, the detection signal is digitized via an ADC, resulting in a multi-frequency superimposed signal containing defect-related information. DFT is applied at predefined frequency points to decompose the signal into constituent frequency components, calculating the real ($ReX\left[k\right]$) and imaginary ($ImX\left[k\right]$) parts using the following correlation-based formulas [8]:

(2) $$ReX\left[k\right]=\sum_{i=0}^{N-1}x[i]\cos\left(2\pi ki/N\right)$$

(3) $$ImX\left[k\right]=-\sum_{i=0}^{N-1}x[i]\sin\left(2\pi ki/N\right)$$

where *N* is the number of sampling points.

These operations enable the extraction of frequency-specific impedance variations, critical for defect characterization and visualization.

### 2.3. Spectrum Leakage Solutions

The improper application of discrete Fourier transform (DFT) can lead to spectrum leakage, where spectral energy spreads across adjacent frequency bins, causing amplitude and phase measurement errors [6,7]. This phenomenon arises from the DFT’s inherent assumption of infinite signal periodicity: when a finite-length signal segment (truncated from a continuous waveform) is not an exact integer multiple of the signal period, abrupt amplitude changes at the truncation points introduce spurious frequency components, distorting the true spectrum [9].

To address this, the proposed method enforces strict periodicity matching between the signal and sampling grid. Specifically, when the relationship of Equation (4) is satisfied, where M is the number of signal periods within the sampling window, N is the number of sampling points, $F_{in}$ is the input signal frequency, and $F_{s}$ is the sampling frequency, the truncated signal segment becomes a perfect periodic repetition, eliminating discontinuities at the segment boundaries and ensuring that the DFT accurately captures the true frequency components without leakage.

(4) $$\frac{M}{N}=\frac{F_{in}}{F_{s}}$$

**Figure 6:**
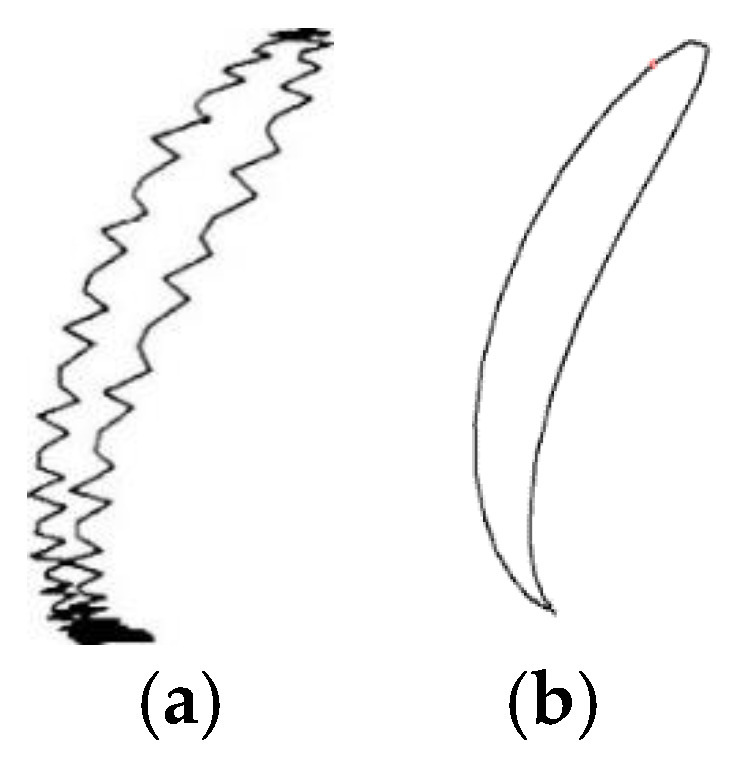
(**a**) Impedance plane plot when spectral leakage occurs; (**b**) normal impedance plane.

Figure 6 illustrates the impedance plane plots under leakage and non-leakage conditions. In Figure 6a, spectral leakage distorts the impedance trajectory, introducing erroneous minor components; in contrast, Figure 6b shows a clean, stable trajectory when the periodicity condition is met, confirming the effectiveness of the proposed strategy.

The experimental results demonstrate that the proposed scheme is capable of thoroughly resolving the spectrum leakage issue stemming from DFT-based signal analysis.

### 2.4. Algorithm Verification

Phase linearity is defined as the consistency of the phase difference between input and output signals across the full 0–360° phase range. A high-performance algorithm should maintain a linear phase response, minimizing phase distortion that could mislead defect evaluation.

**Figure 7:**
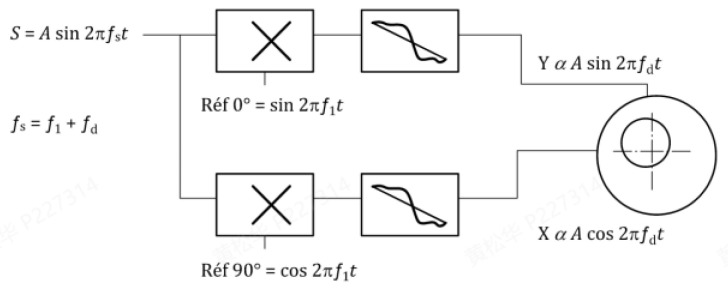
Demodulation block diagram.

The frequency beat method is employed for verification [10], leveraging the phase relationship between two closely spaced frequencies, namely $f_{s}=f_{1}+f_{d}$. As shown in the demodulation block diagram (Figure 7), when a sinusoidal voltage with a slight frequency offset $f_{d}$ is applied to the eddy current sensor, the demodulation process generates the orthogonal signals *X* and *Y*, modulated by $\cos(2\pi f_{d}t)$ and $\sin(2\pi f_{d}t)$, respectively. Low-pass filtering removes high-frequency components, leaving baseband signals for phase linearity analysis [11].

Demodulation consists of determining the real and imaginary parts of the signal.

$$S_{x}(t)=A\sin\left[2\pi (f_{1}+f_{d})t\right]\sin2\pi f_{1}\cdot t$$

This can be rewritten as the following Equation (5):

(5) Sx(t)=A2[cos⁡2πfd·t−cos⁡2π(2f1+fd)·t]

$$S_{y}(t)=A\sin\left[2\pi (f_{1}+f_{d})t\right]\cos2\pi f_{1}\cdot t$$

This can be rewritten as the following Equation (6):

(6) Sy(t)=A2[sin⁡2πfd·t+sin⁡2π(2f1+fd)·t]

Low-pass filtering is used in the sensor signal processing algorithm to suppress the second terms in Formulas (4) and (5) which contain the frequency $\left(2f_{1}+f_{d}\right)$ [11].

At the outputs of the signal processing algorithm, two signals X and Y, the amplitude of which is proportional to A, modulated, respectively, by a cosine and a sine at the frequency $f_{d}$, are available.

**Figure 8:**
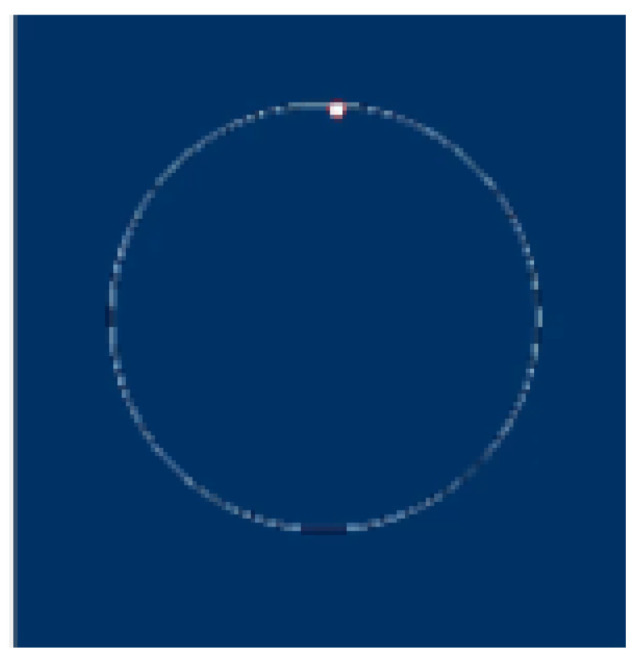
Circle rotates at the frequency of $f_{d}$.

In the case of an ideal signal processing algorithm, these two voltages, applied to an oscilloscope, display on the screen a circle as shown in Figure 8, the radius of which is proportional to A, with the spot rotating at $f_{d}$.

To measure the frequency of the instrument generator, the frequency of the input signal $f_{s}$ is adjusted in order that the spot stops rotating on the screen.

In this case, $f_{1}$ = $f_{d}$.

Based on the above general method, the eddy current signal processing algorithm is verified as follows.

During the verification, the verification software system collects the continuous components of the X and Y output by the signal processing algorithm of the eddy current signal sensor. The values of the ith sample are $X_{i}$ and $Y_{i}$. The continuous component is $X_{av}$ or $Y_{av}$, the average of all samples, and where $X_{irec}=X_{i}-X_{av}$ and $Y_{irec}=Y_{i}-Y_{av}$. The phase angle value for the ith sample is $\varphi_{mi}$ and can be calculated by the following Equations (7) and (8):

(7) $$\varphi_{mi}=\arctan\left(\frac{Y_{irec}}{X_{irec}}\right),\;(X_{irec}\geq 0)$$

(8) $$\varphi_{mi}=\arctan\left(\frac{Y_{irec}}{X_{irec}}\right)+180{}^{\circ},\;\left(X_{irec}<0\right)$$

When all the phase angle values are linearly fitted, the maximum deviation of all phase angles from the fitting line is the phase linearity error.

Linear fitting of these angles shows maximum deviations (phase linearity errors) of ≤0.07° across 20 Hz–1 MHz (Table 1), except for a slight increase at 2 MHz due to ADC sampling rate limitations, confirming excellent phase fidelity.

Experimental results have shown that this approach accomplishes near-ideal spectrum analysis. This high-level of accuracy in phase linearity is crucial for the precise characterization of defects.

### 2.5. Comparative Testing

To validate the proposed digital method, comparative tests were conducted against conventional analog eddy current instruments, focusing on signal quality and defect imaging performance.

**Figure 9:**
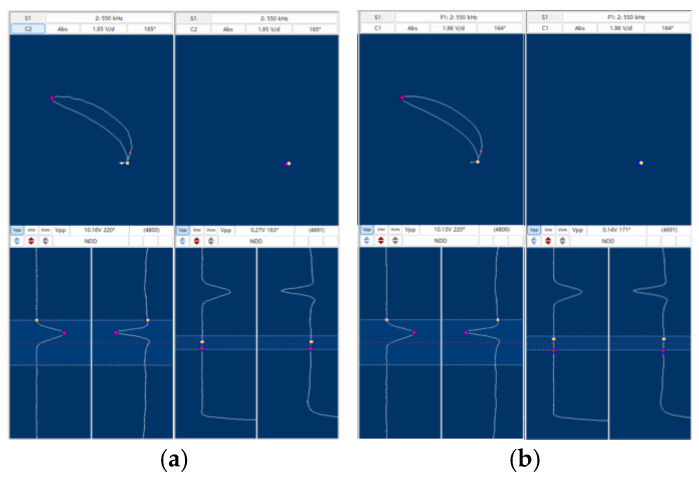
(**a**) Tube test using the analog method; (**b**) tube test using the digital method.

Figure 9 compares the absolute signal waveforms obtained from a calibration tube with through-hole defects at 550 kHz. The analog method (Figure 9a) exhibits significant noise (0.27 V peak-to-peak), while the digital method (Figure 9b) reduces noise to 0.14 V (a 48% improvement). This reduction is attributed to the 24-bit ADC’s wide dynamic range and elimination of analog balancing circuits, which are major sources of electronic noise in traditional systems.

**Figure 10:**
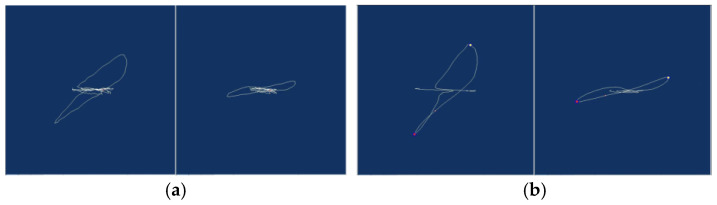
(**a**) Wear test using the analog method; (**b**) wear test using the digital method.

In the wear testing of nuclear heat transfer tubes, the digital method outperforms its analog counterpart in suppressing support structure interference. By mixing 550 kHz and 100 kHz for differential signals and 300 kHz and 100 kHz for absolute signals, the digital approach (Figure 10b) produces smoother, more distinct defect signatures compared to the analog method (Figure 10a). The enhanced signal clarity facilitates easier identification of subtle wear patterns, which is critical for early defect detection in high-stakes nuclear applications [12].

Our experimental results demonstrate that the proposed digital eddy current signal processing method exhibits a superior signal-to-noise ratio compared to its traditional analog counterpart.

## 3. Array Eddy Current Sensor Development

Array eddy current testing offers enhanced detection efficiency and 3D defect visualization for nuclear component inspections [13]. Key challenges include signal integrity maintenance and miniaturization for confined spaces (e.g., 15.4 mm inner diameter tubes).

**Figure 11:**
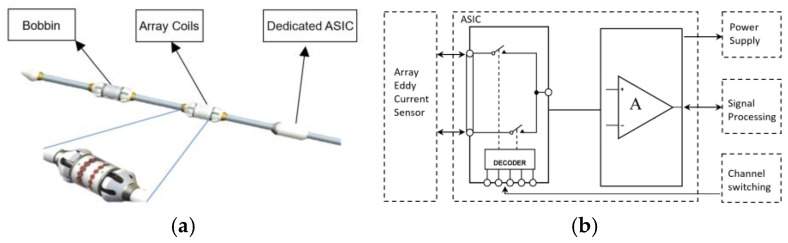
(**a**) Schematic diagram of the structure of the array probe. (**b**) Functions of the ASIC.

A custom 6 × 6 mm ASIC was designed to enable individual coil excitation and parallel signal amplification within the array probes. Figure 11 presents a schematic diagram of the array probe structure, comprising a Bobbin probe, array coils, and a dedicated array signal processing ASIC. The left subfigure illustrates the probe’s internal architecture, while the right subfigure depicts the functional layout of the custom ASIC, highlighting its role in eddy current signal processing.

**Figure 12:**
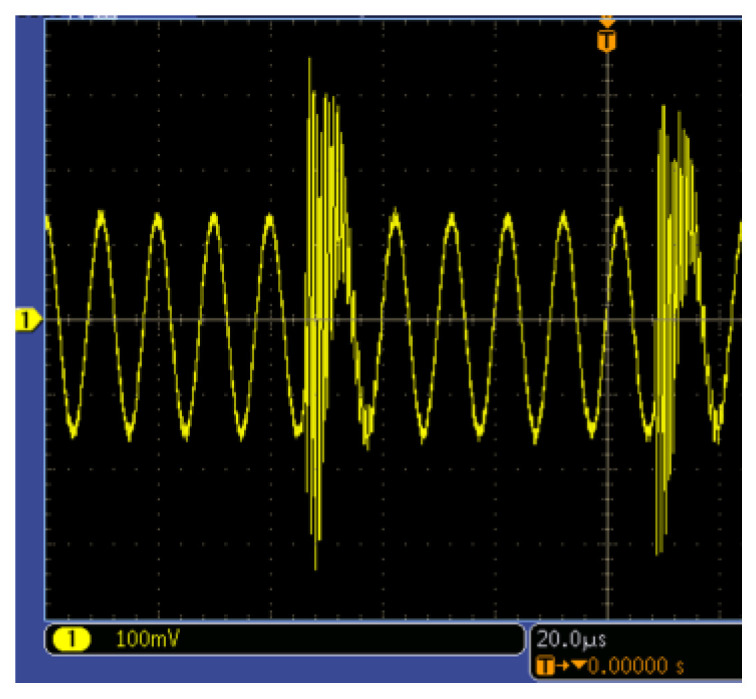
The response of the analog circuit channel switching.

To enable the full acquisition of array coil signals, the ASIC employs precise timing-controlled channel switching. However, transient noise generated during channel transitions introduces artifacts that disrupt the eddy current field and degrade detection accuracy. As shown in Figure 12, analog circuit switching induces a transient response lasting approximately 10 us. To mitigate this, a data-discarding strategy is implemented: the algorithm excludes 15 us of post-switching data.

The array sensor’s operational frequency range is determined by the inspection speed (400 mm/s) and resolution (1 mm), requiring 12 timeslots to cover the target area. This results in a timeslot duration of 1/4800 ≈ 208 us. After accounting for noise exclusion (15 us), the effective acquisition window is 193 us. To meet the signal-to-noise ratio requirement, the algorithm mandates at least 10 signal cycles per timeslot, leading to a maximum allowable signal period of 19.3 us (corresponding to 51.8 kHz). Thus, the minimum operational frequency for array mode is set to 50 kHz, balancing noise suppression and inspection speed.

The sensor system integrates a three-dimensional (3D) visualization algorithm with spatiotemporal synchronization, leveraging structural information of the inspected object and data timing coherence. This fusion enables automatic defect localization, size measurement, and 3D imaging of detection results, critical for interpreting complex defect geometries in nuclear components.

**Figure 13:**
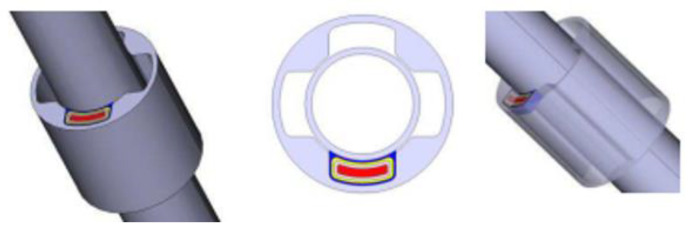
Schematic diagram of quatrefoil tube support plates.

The custom array sensor was deployed during Daya Bay Nuclear Power Plant’s D221 overhaul to assess sludge clogging in quatrefoil tube support plates (Figure 13).

**Figure 14:**
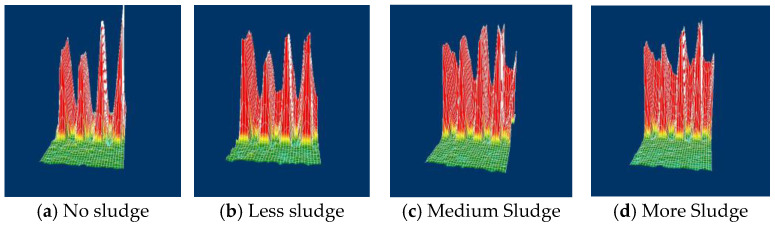
3D display of sludge clogging rate measurement of quatrefoil tube support plates.

At 50 kHz, the system generated 3D amplitude maps (Figure 14) where the horizontal axis represents the tube’s circumferential position, and the vertical axis denotes signal amplitude. In the no-sludge reference (Figure 14a), four high-amplitude signals correspond to the quatrefoil structure and tube interface. The images in Figure 14b–d illustrate varying sludge accumulation levels, with reduced signal amplitude reflecting increased the obstruction in support plate holes.

Our field results demonstrate the following:

1. Stability: The detection system operated reliably, with eddy current array data exhibiting a high signal-to-noise ratio, enabling clear differentiation between defect severities.
2. Visual Clarity: The 3D C-scan maps intuitively displayed sludge distribution in both circumferential and axial dimensions, facilitating the rapid assessment of deposition patterns.

The proposed digital framework outperforms analog systems—prone to impedance mismatches and noise—by delivering consistent, interpretable results across 50 kHz–1 MHz. Its adaptability, paired with a miniaturized ASIC, enables robust high-precision inspections in complex nuclear environments.

## 4. Noise Mitigation Strategies

In nuclear power plant inspections, eddy current probes are susceptible to multiple noise sources, including power frequency interference, electromagnetic coupling, probe vibration, and power supply fluctuations. While grounding and electromagnetic shielding effectively mitigate low-frequency and static noise, dynamic noise induced by probe vibrations poses a significant challenge. Such vibrations introduce high-frequency random fluctuations into the signal, distorting the eddy current response and potentially masking subtle defect signatures, which may lead to inspection errors.

### 4.1. Vibration-Induced Noise and Gaussian Filter Design

Vibration-induced noise manifests as high-frequency fluctuations that disrupt the smoothness of eddy current signals. When these fluctuations exceed a critical threshold, they obscure genuine defect signals, increasing the risk of missed detections. To address this, a Gaussian filter is employed to suppress high-frequency noise while preserving low-frequency defect-related information. The Gaussian filter operates by convolving the signal with a Gaussian kernel, which weights adjacent data points according to a normal distribution. The one-dimensional Gaussian function is defined as in the following Equation (9):

(9) $$G(x)=\frac{1}{\sqrt{2\pi }\sigma }e^{-\frac{x^{2}}{2\sigma^{2}}}$$

where σ controls the width of the kernel, determining the extent of smoothing. In practice, each data point is replaced by a weighted average of its neighborhood, with closer points contributing more significantly [14].

### 4.2. Experimental Validation of Gaussian Filtering

**Figure 15:**
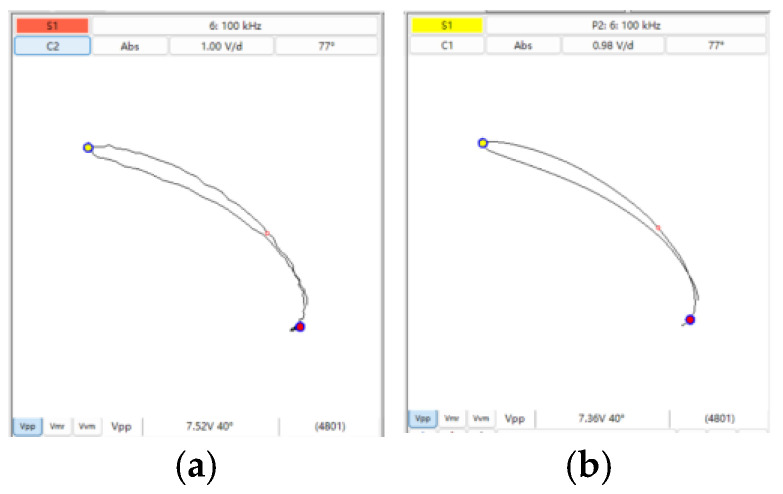
(**a**) Original signal of conventional sensor. (**b**) Gaussian filtered signal.

Figure 15 demonstrates the effect of Gaussian filtering on a conventional eddy current signal. The original signal (Figure 15a) exhibits high-frequency noise spikes, while the filtered signal (Figure 15b) shows significantly reduced fluctuations. This noise reduction enhances the visibility of subtle impedance variations indicative of defects.

**Figure 16:**
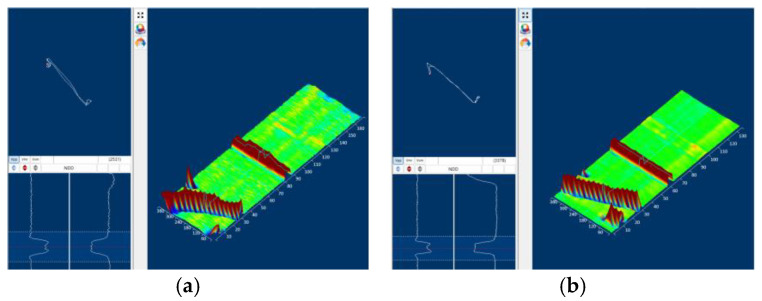
(**a**) Original signal of array sensor. (**b**) Gaussian filtered signal of array sensor.

For array sensors, the Gaussian filter is applied to 3D data arrays, addressing both temporal and spatial noise. Figure 16 compares the raw and filtered signals of an array probe: the unprocessed data (Figure 16a) contain prominent high-frequency artifacts, whereas the filtered result (Figure 16b) presents a clean, artifact-free 3D image, enabling clearer visualization of defect distributions in both the circumferential and axial directions.

### 4.3. Limitations and Future Directions

While Gaussian filtering effectively mitigates vibration-induced noise, it is not universally applicable to all noise types encountered in eddy current testing. Low-frequency noise from electromagnetic interference or temperature drift requires complementary techniques, such as adaptive filtering or frequency-domain notch filters. Future research will focus on developing hybrid noise reduction strategies that combine Gaussian filtering with machine learning-based algorithms to address diverse noise mechanisms.

The results highlight the importance of tailored noise mitigation in maintaining signal integrity for reliable defect characterization. By systematically addressing high-frequency vibrations through Gaussian filtering, the proposed method ensures that eddy current signals remain interpretable, even in harsh nuclear environments with significant mechanical disturbances.

## 5. Application Testing

Extensive field deployments of the proposed eddy current sensor system have demonstrated its effectiveness in nuclear power plant inspections, particularly for heat transfer tube integrity assessment. The digital signal processing framework, combined with the custom array sensor, enabled precise detection of diverse defects, including flow distribution baffle (FBC) wear, support plate (TSP) wear, foreign object-induced damage, and denting.

**Figure 17:**
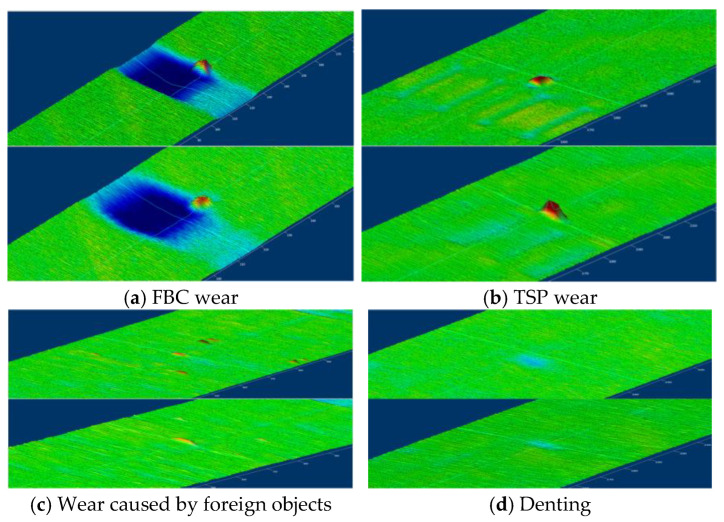
Array eddy current sensor 3D test results.

Figure 17 illustrates 3D imaging results from typical inspections, where defect locations and sizes are clearly visualized through color-coded amplitude maps. For example, FBC wear appears as localized high-amplitude regions due to material loss, while TSP wear manifests as uniform signal attenuation caused by repetitive mechanical stress. These visualizations facilitate rapid defect characterization, reducing interpretation time by 30% compared to traditional 2D signal analysis methods.

## 6. Conclusions

This study presents a robust framework for eddy current non-destructive testing (NDT) in nuclear components, integrating DFT-based signal processing, hardware optimization, and array sensing to address critical challenges in defect characterization. The study’s key innovations include the following:

1. DFT spectrum leakage mitigation: By enforcing strict periodicity matching ($M/N=F_{in}/F_{s}$), the proposed method eliminates spectral distortion, achieving phase linearity errors of ≤0.07° across the 20 Hz–1 MHz range, which is critical for accurate impedance analysis.
2. Hardware-level noise suppression: The 24-bit ADC architecture removes the need for analog balancing circuits, reducing system noise by 48% and enabling direct, high-fidelity signal acquisition in harsh nuclear environments.
3. Array sensor miniaturization: The 6 × 6 mm ASIC enables 3D defect visualization in narrow-diameter tubes (≥50 kHz frequency adaptability), with Gaussian filtering further enhancing signal clarity by suppressing vibration-induced noise.

Our experimental and field results show that the digital method surpasses traditional techniques in terms of signal quality, defect imaging, and inspection efficiency. Its 3D visualization aids early detection of subtle defects missed by analog systems.

Future research will focus on integrating machine learning for adaptive noise cancellation and developing multi-modal sensing to address nuclear component aging challenges. The proposed framework sets a new precision NDT benchmark, enhancing the safety and reliability of nuclear infrastructure through signal and hardware innovations.

## Figures and Tables

**Table 1:** Phase linearity error at different frequencies.

Frequency	Target Value	Measured Value
20 Hz	0° (<1°)	0.06°
100 Hz	0° (<1°)	0.07°
500 Hz	0° (<1°)	0.06°
1 kHz	0° (<1°)	0.07°
10 kHz	0° (<1°)	0.02°
100 kHz	0° (<1°)	0.03°
500 kHz	0° (<1°)	0.03°
1 MHz	0° (<1°)	0.03°
2 MHz	0° (<1°)	0.15°

## Data Availability

The data and intellectual property rights belong to CGN Inspection Technology Co., Ltd. Any sharing needs to be evaluated and approved by the company.
